# Editorial

**Published:** 1864-06

**Authors:** 


### Editorial.

### A TRIP EAST.

During the latter part of April, we made a flying trip East, to obtain a little respite from severe toil, and to see some of the profession at home, and to look in upon several societies that were to meet about that time.

On our way, we first halted, or were made to halt, at Pittsburgh, and met the Pittsburgh Dental Society on the evening of Tuesday, the 26th of April. There was a full meeting, and all were full of zeal and energy for the work in which they were engaged, and here, just as anywhere else, where men engage in an association, they are accomplishing a great work; they are working together in harmony, just like brothers should; and each one feels within himself the resultant good of such efforts; and we know from what we have seen, that the profession in Pittsburgh and its vicinity will, ere long, bear a different name and character from that which has characterized it in former times. After the regular business of the meeting was finished, the Society adjourned to a rich repast which had been provided. Here, too, it was found that all were workers, as well as in the Society, or at the operating chair. It was a time big with social enjoyment, and we can assure any of our professional brethren who may visit Pittsburgh, that they will find the Pittsburgh Dental Association a lively institution, one that will well repay a visit.

Next day we journeyed on to Philadelphia, and saw many of the brethren; all enjoying health and prosperity. The first annual meeting of the Odontographic Society of Philadelphia took place a few days after we were there, but it was impossible to be there; this we regretted exceedingly. We understand it was a large meeting, and one of great interest. Both the Dental Associations there are in a very flourishing condition, and their influence extends throughout the professional world.

While there we had the pleasure of visiting, accompanied by Professors McQuillen, Kingsbury and Flagg, the Philadelphia Dental College, and to us it was a matter of surprise and pleasure to see how much had been done in so short a time, in the way of shaping up a very efficient institution. It is most convenient in all its arrangements, and very complete in its equipments, and the work here accomplished has not been done without a large expenditure of time, labor and money.

This institution, in all that pertains to it, certainly has the elements of success.

On Thursday, the 28th, we went to Wilmington, Delaware, to meet with the Delaware Dental Association; upon arriving we found the members convened and at work. This is a live Association and promises much good for the profession in that portion of the country. They changed the name of the Society to the "Peninsular Dental Association," and to meet quarterly instead of monthly. This was done that members of the profession outside of the State might become members of the Society.

The members are all heartily engaged in this work. A very interesting essay was read by the President, Dr. S. Marshall, and the discussions were of an interesting character. This Society will be fully represented at the next National Association.

From this we went to New York, and there on the evenings of the 29th and 30th, were present at the meetings of the Brooklyn Dental Association and the New York Society. There were about fifty members present at each meeting. Each Society meets every two weeks, and they alternate so that every week there is a meeting, and always largely attended and full of interest. It is very gratifying indeed to see such harmony and good feeling as here exists; we neither saw nor heard a jar in all their operations. In these meetings there are always papers read of interest, and lively discussions.

These Societies have united their efforts in the work of getting up, organizing and establishing a Dental Institute, or College. They are at work in such a manner as will produce great results. Each Society has appointed a committee for this purpose, and they are working together. And if their present plans are executed they will doubtless have one of the best, if not the best institution of the kind in the world. They have the men and the means to do it, and they are disposed to use both. We know of no place where greater harmony prevails in the profession than in New York. This is all the more remarkable and pleasant from the fact that formerly it was far otherwise. This is due to associated effort. We think there is no place in the world where such rapid progress is being made in the practice of our profession, as in the city of New York and vicinity. With the present movement of things, it will not be long till almost every dentist there will be a first class operator; we have reference to those of course who are availing themselves of the opportunities that are before them. They will be fully represented at Niagara in July next.

From New York we went to New London, Conn., where, on the 2d of May, we had the privilege of being present at a preliminary meeting of the Dentists of that city and vicinity, for the purpose of organizing a Dental Society, and it was very pleasing and interesting to witness the spirit manifested there. Those who scarcely ever met before were here cordially together, entering upon a work that we trust and feel assured will not soon terminate. A committee was appointed to prepare a Constitution and By-Laws, and another meeting for effecting the organization was appointed to take place in two weeks; and we expect soon to hear a good report from it.

From this point we went to Lowell, Mass., to be present at the regular meeting of the Merrimac Valley Dental Society, which was held on Thursday, the 5th. There we had the pleasure of meeting a large number of the profession of that part of the country. Here, as elsewhere, there exists the utmost professional harmony and good feeling. This is one of the pioneers, though a young one, in New England. This Society is also working vigorously, and accomplishing great good for the profession. They have their Constitution and By-Laws printed in neat form, a thing which we think every society should do; and we would suggest that all Associations exchange these documents.

They appointed a committee to get up and publish a popular essay, to be distributed by the members to their patients. This we regard as due the profession and the public; and it is desirable that every society should enter upon some such work.

The President read a very excellent paper upon "Professional Etiquette," which is published in this number. This essay will bear several readings. This society gives evidence of being a permanent and vigorous institution. Its influence will permeate the profession in New England.

From Lowell we journeyed to Hartford, to be present at the Meeting of the Connecticut Valley Dental Association, which met on Friday the 6th.

Upon arriving, we found it to be a union meeting of that Society with the New Haven Society, together with a considerable number not members of any association. There was in all quite a large meeting, in which true zeal and interest were manifested.

We should think from what we saw and heard, that they have gone to work with all the zeal and energy of new converts; they are certainly taking hold in earnest. We have one suggestion to make to our New England brethren: it is, that they form a greater number of local societies, and meet far more frequently. The greatest interest and efficiency is only attained by frequent intercourse.

Every such city as Hartford, Springfield and New Haven should have a Dental Society, and meet certainly not less than once a month, and more frequently would be better. From what we saw we will predict for the profession in New England an unprecedented progress both in rapidity and extent, during the next five years. We do not mean by this to insinuate that they are in the rear now, but they possess the elements of progress in a large degree, and they are arousing to the importance of employing them.

We intended visiting two or three other societies in our trip but found we were compelled to return home from Hartford.

It is a matter of great gratification indeed to witness such a spirit as now pervades the profession. There seems to be a thorough awakening to the importance of effort for professional progress. There is a better appreciation of our science and art, both by the profession and the people, than heretofore. The number of faithful, earnest and industrious workers is increasing in the profession. And notwithstanding the growth of our profession has been wonderful, and to many astonishing, yet we hesitate not to predict for it a far greater and more rapid development in the immediate future. T.

### GONE TO THE WAR.

Our co-editor and friend, Dr. G. WATT, has entered the service of our country, in the capacity of Surgeon to the 154th O. N. G., to be gone a hundred days. He is now stationed at New Creek, W. Va., said to be a very good place for guerrillas.

By this arrangement, the readers of the *Register* will suffer loss. We all suffer from this war; but we make this timely announcement that our readers may know what's the matter for the next three months.

We trust he will return at the end of his term safe, much strengthened and invigorated by the change. T.

### POLISHING POWDERS.

We have for sometime been using a preparation consisting of calcined buckhorn, for polishing fillings, which accomplishes the work better than any thing we have ever employed. It cuts rapidly, and leaves a fine surface; will polish the dentine as well as the filling. It is furnished by S. S. White, in the form of powder in boxes, or packages, and on tape, after the manner of corundum tape. It is equally good for polishing gold plates, and we presume rubber. We advise all to try it, and if it works as well with others as it has with us, we are certain they will not consent to be without it. T.

### PRESENT CONDITION OF AN OLD CASE.

Within the last few days, we have had the pleasure of seeing the gentleman for whom we treated a severe case of alveolar abscess, an account of which is given in full in the *Register* of May, 1861. The case is now in a better condition than we could have anticipated. The gentleman's health is very good; the new alveolus is firm and healthy, sustaining the teeth as firmly, if not more so, than they were ever before. The gums are in a perfectly healthy condition, and present a beautiful appearance, Taken altogether, this case is as successful as any one could desire; notwithstanding it was in every aspect, both so far as local and systemic conditions were concerned, most unfavorable and discouraging in the beginning. We have, in this case, encouragement to faith, labor and zeal in future cases. T.

